# Niche Partitioning in Three Sympatric Congeneric Species of Dragonfly, *Orthetrum chrysostigma, O. coerulescens anceps*, and *O. nitidinerve*: The Importance of Microhabitat

**DOI:** 10.1673/031.013.7101

**Published:** 2013-07-15

**Authors:** Rassim Khelifa, Rabah Zebsa, Abdelkrim Moussaoui, Amin Kahalerras, Soufyane Bensouilah, Hayat Mahdjoub

**Affiliations:** 1 Département d'écologie et du génie de l'environnement, Université 08 Mai 1945, Guelma 24000, Algérie; 2 Laboratory of Electrical Engineering, Guelma (LGEG), Université 08 Mai 1945, Guelma

**Keywords:** breeding behavior, habitat preferences, niche overlap

## Abstract

Habitat heterogeneity has been shown to promote co-existence of closely related species. Based on this concept, a field study was conducted on the niche partitioning of three territorial congeneric species of skimmers (Anisoptera: Libellulidae) in Northeast Algeria during the breeding season of 2011. According to their size, there is a descending hierarchy between *Orthetrum nitidinerve* Sélys, *O. chrysostigma* (Burmeister), and *O. coerulescens anceps* (Schneider). After being marked and surveyed, the two latter species had the same breeding behavior sequence. Knowing that they had almost the same size, such species could not co-occur in the same habitat according to the competitive exclusion principle. The spatial distribution of the three species was investigated at two different microhabitats, and it was found that these two species were actually isolated at this scale. *O. chrysostigma* and *O. nitidinerve* preferred open areas, while *O. c. anceps* occurred in highly vegetated waters. This study highlights the role of microhabitat in community structure as an important niche axis that maintains closely related species in the same habitat.

## Introduction

Processes that determine species' coexistence and exclusion are central topics in community ecology. According to Hutchinson (1957), an ecological niche is an n-dimensional hyperspace where each axis (dimension) represents a resource or an environmental condition. A common assumption is that sympatric species with a similar phenotype tend to reduce conflict by occupying different niches (Colwell and Fuentes 1975). The more the overlap in species' niche, the stronger the competitive interactions between them (Hardin 1960). Of course, given the many aspects of a niche, measuring a species' entire niche is impossible. However, Schoener (1974) proposed microhabitat, diet, and temporal activity as the three most important niche axes, and subsequently a large range of empirical data regarding these components in several animal groups has become available, for example in mammals (Doniol-Valcroze 2008), birds (Salewski et al. 2002; Parra et al. 2004), reptiles (Metzger et al., 2009), amphibians (Kuzmin 1990; Behangana and Luiselli 2008), and insects (Heinrich 1976; Coderre 1987; Crowley and Johnson 1982; Gilbert et al. 2008; Prieto and Dahners 2009; Venner et al. 2011).

Another determining factor in community structure is body size. Resource use (e.g., prey) depends on the body size of predators and often determines competition with heterospecifics. As a result, body size is not randomly distributed among species within the same community, but rather it is evenly spaced (Hutchinson 1959). In other words, there is a critical size similarity threshold that allows species coexistence (Horn and May 1977). Otherwise, interspecific competition would act like a limiting force inducing regu-lar exclusions between morphologically similar species. Brown and Wilson (1956) stated that such species would adopt character displacement (morphological differences between closely related species coexisting in the same area) as a solution when they live in sympatry. Moreover, predation and competition for resources have also been shown to determine species coexistence by specifically excluding groups of species (McPeek 1989, 1990). For example, within the genus of *Enallagma,* some species were found resident in fishless lakes while others were only found in lakes containing fish in many North American lakes (Johnson and Crowley 1980; Crowley and Johnson 1982; McPeek 1989, 1990).

Adult odonates, which are usually territorial during the breeding season, are a good biological model for inter- and intraspecific competition studies (Moore 1964). Usually, mature males defend a specific territory against intruders and thus attempt to dominate access to females (Corbet 1999). Given the limited availability of suitable breeding habitats and females, territories can be considered as a limited resource for which territorial males regularly compete during this period. Usually, the intensity of intra- and interspecific competition is not equal, with the former being the stronger. However, it can almost be the same when species show similar morphological characteristics, like it has been shown for some species of Coenagrionidae (Moore 1964; Beukema 2004), Calopterygidae (De Marchi 1990; Nomakuchi and Higashi 1996; Tynkkynen et al. 2004; 2005), and others.

In odonates, many congenerics have apparently similar habitat requirements and usually live in sympatry. These species are a good model to study interspecific competition and niche partitioning. In a recent study on the inventory of adult odonates dwelling in the Seybouse watershed in northeast Algeria, three species of *Orthetrum,* the yellow veined skimmer, *Orthetrum nitidinerve* Sélys (Anisoptera: Libellulidae), the Epaulet skimmer, *O. chrysostigma* (Burmeister), and the keeled skimmer, *O. coerulescens anceps* (Schneider), were usually found sharing the same locality during their flight period (Khelifa et al. 2011). In a context of ecological niche, these three species are quite similar morphologically and behaviorally, share the same resource (territories in the breeding area that increase the breeding success of males), live in the same habitat, and at the same time their coexistence is almost, or totally, impossible for many theorist biologists (Volterra 1928; MacArthur and Levins 1964; Levin 1986; and others). However, if these species co-occur in a natural environment, they must necessarily have, to some extent, separate niches by at least one axis.

**Figure 1.**
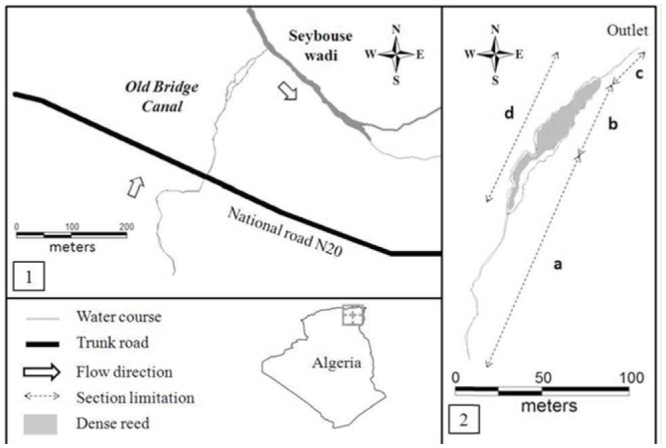
Presentation of the study site. 1. Map of the Old Bridge canal. 2. Map of the sample area; a, b, c, and d present the sections sampled. Section a and c were open areas while section b and d were covered and high vegetated areas. High quality figures are available online.

Odonates have been well-studied in the field of niche partitioning, but most work has been focused on the larval stage (Crowley and Johnson 1982; Dudgeon 1989; Sternberg 1991, Wissinger 1992; Mahato 2000). In the present study, the breeding behavior and microhabitat use of adults of three congeneric dragonfly species, *O. chrysostigma, O. coerulescens anceps,* and *O. nitidinerve,* were surveyed to discover the ecological mechanisms that maintain their coexistence.

## Materials and Methods

The study was undertaken upstream of the Seybouse River in Northeast Algeria at the Old Bridge canal, which is situated 5 km west of Guelma city (36° 28′ N, 7° 22′ E). The watercourse is 450 m long, and has an average depth and width of 7 and 120 cm respectively. At its lower part, it splits in two streamlets flowing on both sides of a dense reed for about 80 m, which then meet at 30 m from its outlet to the Seybouse River (Figure 1). To our knowledge, the canal has never been used for irrigation. The dominant vegetation at the edge of the canal was exclusively shrubs of oleander, *Nerium oleander* L. (Gentianales: Apocynaceae), at its upper region, but the rest was dominated by cattail, *Typha angustifolia* L. (Poales: Typhaceae), sea rush, *Juncus maritimus* Lamark, and knotgrass, *Paspalum distichum* L. near its outlet. In addition to the three study species, other species of Odanata were also present, including the copper demoiselle, *Calopteryx haemorrhoidalis* Vander Linden, the Iberian bluetail, *Ischnura graellsii* Rambur, featherleg, *Platycnemis subdilatata* Sélys, small red damselfly, *Ceriagrion tenellum* De Villers, and Mediterranean bluet, *Coenagrion caerulescens* Fonscolombe.

### Breeding behavior observations

Daily observations were made on the breeding behavior of the three skimmer species from 20 June to 25 July 2011 in the morning (10:30– 12:30) and afternoon (13:30–15:30). Mature adults were individually marked on their right hind wing with a permanent marker and followed from an observation point at a distance of 3 m. At this position, the observer can both easily detect the conspicuous marks and avoid any disturbance on active individuals. The classification of Sakagami et al. (1974) was followed to place each species in its breeding behavior category (Table 1). Quantitative data (the duration of each behavior component) were not reported here because they were not thought to have a crucial role in the interspecific competition intensity. Every dragonfly species has a complete breeding behavior sequence that starts with the encounter of the two sexes and ends with oviposition. The purpose of this study was only to highlight potential qualitative (presence or absence) similarities in the breeding behavior components of the three congeneric species. Since they were all territorial, and resembled one another in coloration (for the three species, mature males and females are blue and brownish-yellow, respectively), it was assumed that an individual of a skimmer species displaying a particular behavior (like oviposition in females) within a breeding area is more likely to be interfered or intercepted (when it is a female) by a male belonging to a different species and have a similar behavior in its species ethogram. Therefore, the more the similarities in breeding behavior sequence between species, the stronger the interference competition.

**Table 1.**
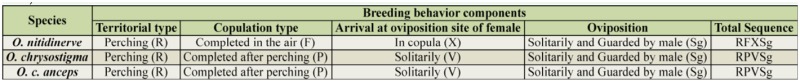
Breeding behavior description of the three dragonfly species. Lowercase in acronyms refers to transitional type (Sakagami et al. 1974).

**Table 2.**
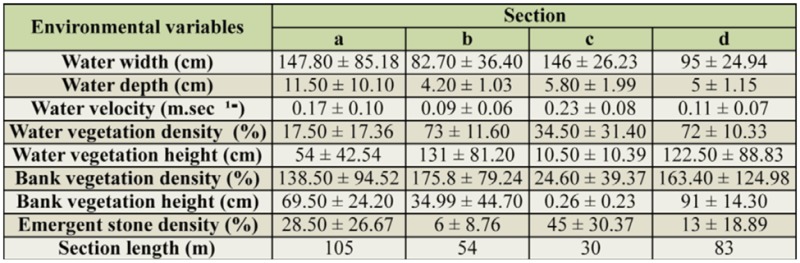
Mean ± standard deviation values of abiotic factors for the four sections sampled.

### Body size measurements

Mature skimmers (of the three skimmer species) were captured by a hand net, marked individually by a permanent marker on the right hind wing and thorax, and some of them were measured (the total body length from the head to the end of the abdomen including cerci and the left hind wing length) by a digital caliper to the nearest 0.01 mm.

### Microhabitat survey

The three skimmers were not common at the upper part of the watercourse, which was dominated by a large population of *C. haemorrhoidalis.* Therefore, our study was only focused on downstream of the canal, limited to approximately 200 m, and divided to 4 sections according to the watercourse's physical characteristics (Table 2) and especially vegetation cover. Section a and c were open areas, while section b and d were highly vegetated (covered) areas. Some abiotic factors were randomly taken from 10 points within each section, recording the water width, depth, velocity, emergent vegetation height and density, bank vegetation height and density, and emergent stone density. A quadrat (1 m^2^) was used to estimate densities. The flight period of *O. chrysostigma* and *O. nitidinerve* started in late May, while *O. c. anceps* adultsstarted to be observed only in early June. The investigation was initiated when the three species became reproductively active. The spatial distribution of the three skimmers was surveyed daily from 10:00 until 16:00 from 12 June until 28 July 2011 by slowly walking along the canal and hourly recording the number of individuals (males and females) of each species within each section. Sex was not considered for further analysis. Immature individuals were not included in the analysis, because they could bias the results because mature adults could easily influence their distribution. For further analysis, only the maximum number recorded within a day was taken (usually at 12:00).

### Translocation experiment

*O. c. anceps* was the smallest species within the three studied skimmers, and one might think that its distribution could be substantially affected by the two larger ones inducing its exclusion from its preferred (micro-) habitat. To examine this, removal and translocation experiments were done in section c during 3 consecutive days (26–28 July). This section was chosen because its small size enabled a quite perfect control (see Figure 1.2), and also no skimmer individual would enter the section coming from the river through the canal outlet (Figure 1). This period was also chosen because the numbers of the two larger species had begun to decrease in late-July, which was advantageous for the experiment. First, each skimmer was captured and kept in a cage (they were released after the experiment). Then, 30 marked mature males (10/day) of *O. c. anceps* coming from section b and d were translocated to section c. It is important to point out that individuals were not released in the air because that induced their instant dispersal out of the section. Instead, they were taken by the wings, deposited carefully on a plant support in the middle of the section, and were released after they were able to grasp the vegetation. By doing so, individuals tended to stay in the same position. After 15 minutes (during which few skimmer individuals entered the section), the host section was first checked and then the 3 remaining sections were intensely searched for potential marked individuals, which were recorded in their respective position.

### Statistical analysis

**Figure 2.**
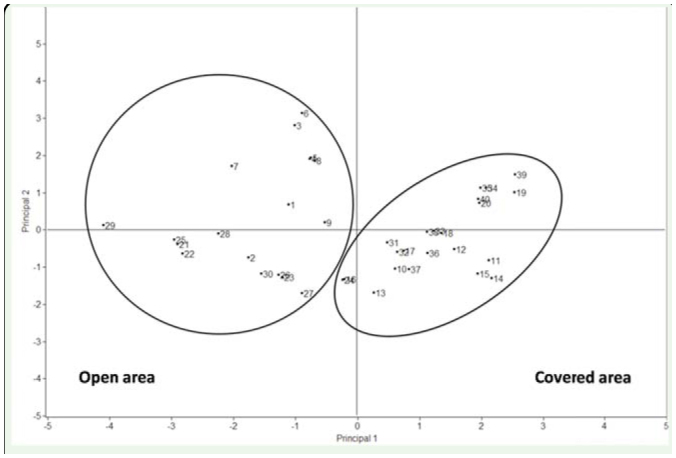
Position of the all samples in a principal component space using eight environmental variables (water width, water depth, water velocity, emergent vegetation density, emergent vegetation height, bank vegetation density, bank vegetation height, emergent stone density). Samples 1–10 were taken from section a, 11–20 from b, 21–30 from c, and 3 1–40 from d. High quality figures are available online.

Most statistical analyses were computed by SPSS 17.0. To analyze the spatial distribution data, data of sections with similar physical and environmental characteristics (Table 2) were pulled together after a principal component analysis (Figure 2, Table 3). In other words section a with section c, and section b with section d for open and covered areas, respectively. To compare the number of individual adults counted in open versus covered sections, a Mann-Whitney test was performed using an alpha-value of 0.05. A Kruskal-Wallis test was carried out to illustrate the difference of body and hind wing lengths between species (alpha-value = 0.05), and a Dwass—Steel—Chritchlow—Fligner post hoc test was computed for pair-wise comparisons (Critchlow and Fligner 1991). EcoSim 7.0 software (Gotelli and Entsminger 2001) was used for the analysis of niche and size overlap. Pianka's index was used to calculate the niche overlap for each pair of species using microhabitat use data (Pianka 1973). Regarding the size overlap analysis, the null model hypothesis supposes that there is an even interspecific spacing in body size of adjacent species, i.e., the smaller the segment length (δ^2^), the more homogeneity in size ratios between coexisting congeneric species (Gotelli and Illison 2002). Variance in segment length metric, the Log uniform distribution, and Log transformed data were used.

**Table 3.**
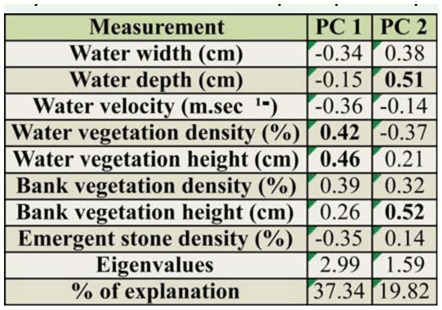
Principal Component Analysis for the two principal components retained for interpretation. The table presents Eigenvectors (correlations) for each abiotic factor (values greater than 0.40 are in bold), eigenvalues, and percentage of explanation. The two principal components with eigenvalues greater than I were retained for interpretation. 95.16% of the data variability was explained by the association of the two axes. Water velocity and emergent stone density were negatively correlated with the first principal component, while water vegetation and water vegetation height were positively correlated with the same axis. Water depth and bank vegetation density were positively related to the second principal component.

## Results

### Description of the breeding behavior

During the study period, observation were made for 175 hours over 35 days, and 39, 31, and 19 complete breeding behavior sequences of *O. nitidinerve, O. chrysostigma,* and *O. c. anceps* were observed, respectively. The low observation number of the last species was due to its tendency to copulate at high vegetation cover, which made observation difficult. Also, a complete sequence of the reproductivebehavior was not easy to survey, due to disturbances produced by conspecifics, other skimmer species, or cattle crossing the canal. A total of 102 individuals of *O. nitidinerve* (58 females, 44 males), 34 *O. chrysostigma* (16 females and 18 males), and 53 *O. c. anceps* (19 females and 34 males) were individually marked during our survey.

Interspecific interferences were frequent between males of *O. nitidinerve* and *O. chrysostigma.* Contests between *O. nitidinerve* and *O. c. anceps* were less common. However, males of *O. chrysostigma* and *O. c. anceps* rarely interacted. Attempts for heterospecific pairing were observed between all species. Pre-copulatory tandems between *O. nitidinerve* males and *O. chrysostigma* females and between *O. chrysostigma* males and *O. c. anceps* females were detected. Although pairs tried to form the copulatory wheel, interspecific copulation was never noted.

A summary of the reproductive behavior of the three species is presented in the Table 1. It was noticed that *O. chrysostigma* and *O. c. anceps* had the same complete behavioral sequences, and *O. nitidinerve* behaved in the same way before copulation but substantially different just after. All the study species were territorial. Males remained perched on a support, expressing strong agonistic behavior toward any skimmer male (irrespective of species) approaching their territory. Once the female arrived, males rapidly went in her direction, intercepted her in the air, and formed the pre-copulatory tandem. This state did not last a long time, just few seconds (sometimes 1 sec) before the copulatory wheel establishment. However, pre-copulatory tandems were sometimes recorded perched on a support in the same state for some minutes. Observations from marked individuals revealed that those couples were the ones usually chased by other males. If copulation is considered to begin when the copulatory wheel occurs, then for the three species the copulation started in the air but the majority of its duration was spent at a perching site.

**Figure 3.**
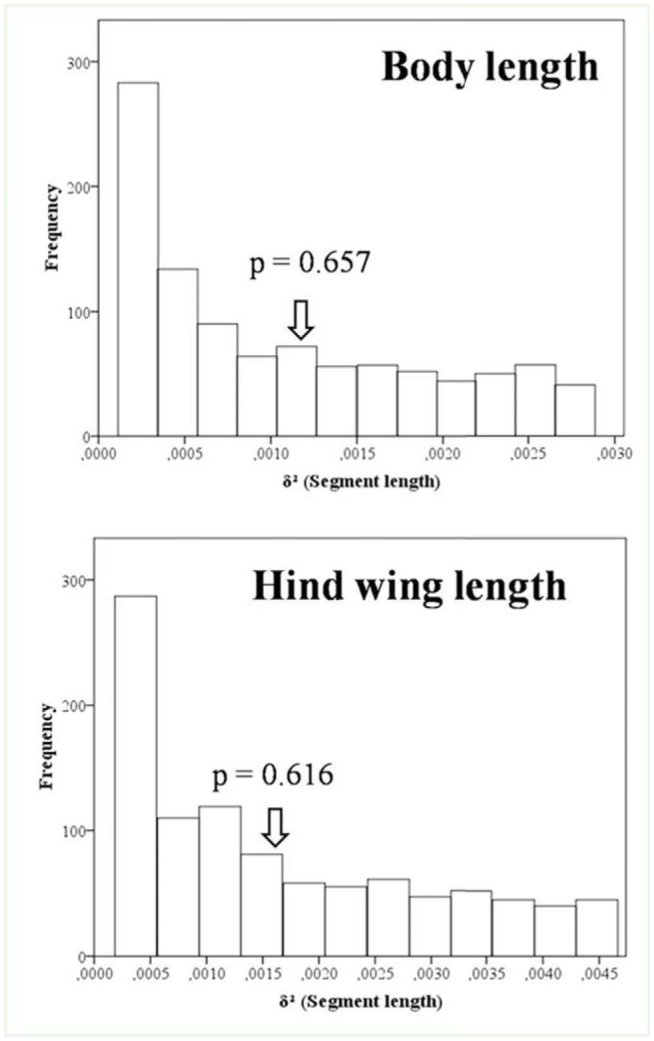
Body size overlap patterns for the body and hind wing length of dragonflies. Frequencies of simulated variance in segment length (δ^2^) are shown in the histograms. They give a good picture on whether the size ratios between adjacent species are stable or not. Arrows point out the observed variances. Values beside arrows are the tail probabilities coupled to the observed variances. High quality figures are available online.

After copulation, the male *O. nitidinerve* went to the oviposition site in copula, hovered above it with some up-and-down movements at 20 cm from the water surface, released the female, and undertook few patrols around her before perching next to her for guarding. The female immersed her abdomen in the water,stayed in an immobile state by holding a support, and wriggled her abdomen from time to time. Strings of eggs produced by the female were fixed to the support surface, which was either a plant stem or a stone. On the other hand, the *O. chrysostigma* and *O. c. anceps* male released the female at the perching site and then patrolled and ended up perched next to her. The female, on the other hand, rested alone at the perching site for a while and then headed to the oviposition site to lay her eggs, followed closely and synchronously by the male. The oviposition behavior of both species was completely different from *O. nitidinerve.* She beat the end of her abdomem many times on the water surface in flight and regularly changed her laying points.

### Size overlap

The size of the three species was significantly different (Kruskal-Wallis test for body and hind wing length respectively: *x*2 = 75.20, df = 2, *p* < 0.0001; *x*2 = 73.73, df = 2, *p* < 0.0001) (Table 4). Multiple comparison revealed that the size of each species differed significantly from the others (body length: *O. nitidinerve* > *O. chrysostigma*: *p* < 0.0001; *O. nitidinerve* > *O. c. anceps*: *p* < 0.0001; *O. chrysostigma* > *O. c. anceps*: *p* < 0.0001; hind wing length: *O. nitidinerve* > *O. chrysostigma*: *p* < 0.0001; *O. nitidinerve* > *O. c. anceps: p* < 0.0001; *O. chrysostigma* > *O. c. anceps*: *p* < 0.0001). The two morphological parameters were used to measure the size overlap between the three species. For both traits, there were quite large variances of segment lengths, indicating that although the three species differed significantly in size, the ratios of adjacent species did not appear more evenly spaced than expected by chance (Figure 3). Table 4 shows that *O. nitidinerve* was the largest species and that *O. c. anceps* was the smallest. *O. chrysostigma* and *O. c. anceps* were the most similar skimmers, differing only by approximately 2 mm, while the larger *O. nitidinerve* exceeded them by at least 6 and 8 mm respectively in both body and hind wing length (Table 4).

**Table 4.**
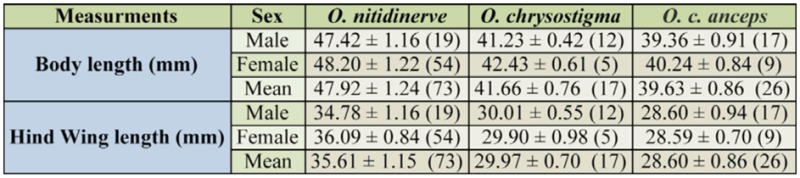
Body and hind wing length of the three skimmer species. Values represent the mean ± standard deviation. Value between brackets is sample size.

### Spatial distribution

**Figure 4.**
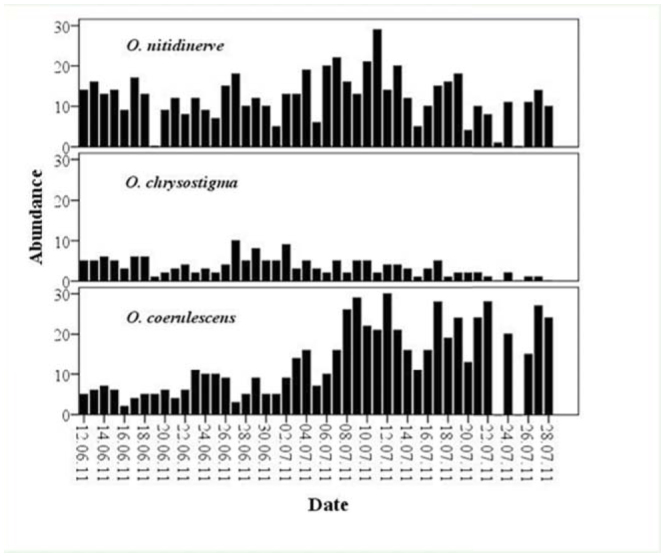
Abundance of the three species of dragonfly during the study period. Bars represent the number of both sexes. High quality figures are available online.

Figure 4 reveals that *O. chrysostigma* was the least abundant species (mean of maximum per day = 1.71), never exceeding 10 individuals. The two other species, clearly more abundant with 6.10 for *O. nitidinerve* and 6.64 for *O. c. anceps,* showed a substantial increase in July, especially for the latter. Abrupt and usually synchronous decreases in the three species numbers were due to bad weather conditions. For example, on 25 June, no skimmer individual was recorded because of heavy wind.

**Figure 5.**
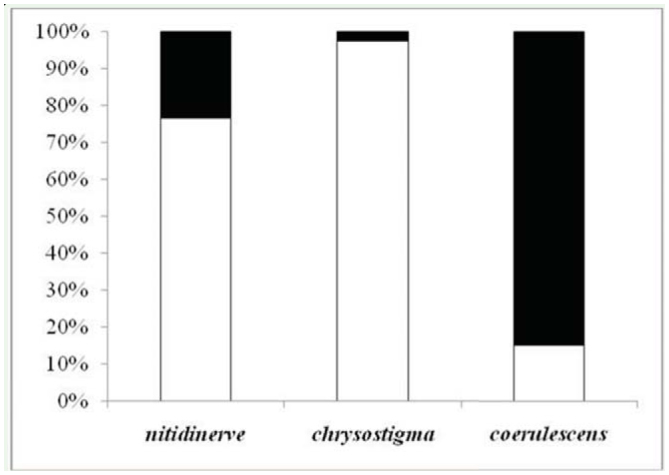
Utilization of microhabitats (open and covered) by the three skimmer species. Open and filled bars refer to open and covered microhabitats, respectively. High quality figures are available online.

The spatial distribution of the three species was not homogeneous along the two microhabitat types (U = 258, *p* < 0.0001; U = 93, *p* < 0.0001; U = 117.5, *p* < 0.0001, for *O. nitidinerve, O. chrysostigma,* and *O. c. anceps,* respectively). Figure 5 presents the adult proportions of the three skimmers recorded in the two microhabitats. It shows the occurrence of *O. chrysostigma* and *O. nitidinerve* in open areas, and *O. c. anceps* in covered ones. It may inferred that the former species was exclusively confined to open areas, since only 4 individuals were observed in covered sections during the whole study period. Similarly, *O. nitidinerve* predominately occurred in open sections, but a quite substantial proportion also frequented vegetated ones probably because its high numbers elicited strong intraspecific competition. In fact, a male was often observed chasing another one from his territory, travelling up to 50 m. The loser of the contest usually perched in peripheral sections. On the other hand, *O. c. anceps* was mainly located in covered sections, and its occurrence in open ones was generally due to the presence of some small vegetated parts usually occupied by 1 individual male. Two species, *O. nitidinerve* and *O. chrysostigma,* mainly co-occurred in the same open microhabitat, and one species, *O. c. anceps,* occupied highly vegetated microhabitat.

**Table 5.**
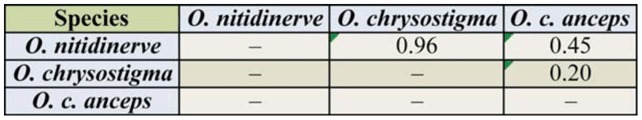
Pairwise niche overlap between the three skimmer species.

### Translocation experiment

The translocation experiment, which moved 30 marked individuals (males) of *O. c. anceps* from covered sections to an open and empty (absence of skimmers) one, showed that no individual remained at the same host section, and 16 returned to the covered ones (three of them were recorded at the same position (territory) where they had been captured). The remaining 14 individuals were not recovered, probably because of their displacement out of the canal. Therefore, it can be inferred that the smaller (*O. c. anceps*) species was not excluded from open areas by the two larger ones, but rather simply preferred areas with high vegetation cover.

### Niche overlap

Niche overlap estimates using Pianka's index are presented in Table 5 for the three species pairs. The lowest niche overlap value (0.20) was between *O. c. anceps* and *O. chrysostigma,* an intermediate one (0.45) was observed between *O. nitidinerve* and *O. c. anceps,* while the highest (0.96) was between *O. nitidinerve* and *O. chrysostigma* (Table 5). Like the spatial distribution revealed, the two former species rarely occupied the same area, whereas the third species pair consisted of skimmers with the same microhabitat preferences.

## Discussion

Our study emphasized the role of habitat heterogeneity in maintaining the coexistence of closely similar congeneric species living in sympatry. The main question was how congeneric species with similar morphology and behavior living in the same system and at the same time could coexist. Reproductive behavior, size overlap, and microhabitat use were investigated to reveal potential biological and ecological isolation between three sympatic congeneric species of dragonflies (genus *Orthetrum*).

Breeding behavior and ecology of the subspecies *O. c. coerulescens* have been well documented (Parr 1983; Buchwald 1989; Miller and Miller 1989; Buchwald and Schmidt 1990; White 2006; White 2008), probably because of its large distribution in the Western Palearctic. Similarly, *O. chrysostigma* was also studied extensively in sub-Saharan Africa (Miller 1983; Schenk et al. 2004; Koch 2005; Koch and Suhling 2005; Suhling et al. 2006). However, data regarding *O. nitidinerve,* a species with a distribution restricted to the Mediterranean basin (Dijkstra et al. 2007), are still lacking (Khelifa et al. 2012).

Similarities in appearance, behavior, and body size support the view that at least two species could not live in the same habitat. The three study species belonged to the *Orthetrum* genus and had broadly the same blue coloration. After surveying them during their breeding season, it was found that two of them, *O. c. anceps* and *O. chrysostigma,* had similar breeding behavior sequences. It has been shown that coloration (Andrew 1966) and flight pattern (Pajunen 1966) affect visual recognition of adult Odonata so that individuals can display predatory, agonistic, breeding, or no behavior accordingly. Waage (1975) showed that two sympatric Calopterygidae, *Calopteryx maculata* and *C. aequabilis,* in North America used wing pigmentation to discriminate between congenerics, but reproductive isolation was not absolute, because interspecific copulation often occurred. The breeding behavior of *O. c. anceps* was similar to that of the subspecies *O. c. coerulescens* reported by Parr (1983), Miller and Miller (1989), and White (2006), matching also that of its congeneric *O. chrysostigma,* which was already investigated by Miller (1983). However, *O. nitidinerve* had a quite different behavior, first described in our study and rarely observed in dragonflies (Corbet 1999).

In terms of interspecific interference, *O. c. anceps* was not totally isolated from the two other species. Open sections also supported small, vegetated patches that could be occupied by an individual mature male. Within a plot of 4 m^2^, for example, the three species maintained different perching sites and agonistic behaviors often took place regardless of size (the smallest could interfer with the largest), as was observed in three coexisting species of *Leucorrhinia* (Singer 1990). Similarly, using marked individuals, interspecific pairing was observed, but copulation was never observed, apparently because males had difficulty maintaining the copulatory tandem. Similar observations were recorded between *Libellula quadrimaculata* females and *L. depressa* males (Paine 1994). It is probable that there is a degree of similarity in anal appendages and female prothorax of the three congeneric species that enabled heterospecific pairing (Pinhey 1963; Lieftinck 1981). In addition, intra- (between males) and intersexual interactions with congenerics suggested a poor recognition between the three dragonfly species. Such mistaken species recognition was also reported between two closely related species of damselflies, *Calopteryx virgo* and *C. splendens* (Tynkkynen et al. 2005).

In Odonata, interspecific interference was shown to exclude conspecifics from foragingareas (Baker 1981) and affect larval habitat use (Suhling 1996; Suutari et al. 2004). Near the Indian Ocean, two arboreal day geckos, *Phelsuma ornata* and *P. cepediana,* shared the same tropical forest (Harmon et al. 2007). Removal experiments of the latter species induced the increase in abundance of the later one, which highlighted the effect of interspecific interactions between these two congenerics. Based on this assumption, our field experiment was performed to investigate potential shifts in resource utilization (microhabitat occupancy) of the smaller species (*O. c. anceps*) after removal of larger congenerics. It showed that *O. c. anceps* preferred highlyvegetated areas, and in contrast to the study on geckos (Harmon et al. 2007), its occurrence in that specific microhabitat was not the result of exclusion by larger species.

The even spacing of body size of species dictated by Hutchinson (1959) was not found in the three species studied when the whole system was considered (excluding habitat heterogeneity), because *O. c. anceps* and *O. chrysostigma,* displaying the same breeding behavior, also had quite similar size. Brown and Wilson (1956) and Hutchinson (1959) concluded that coexistence is not possible in such a case. Even spacing of morphology and body size has been noticed in some insect groups, such as Coleopterans (Brandl and Topp 1985) and Dipterans (Syrphidae) (Gilbert et al. 1985).

Many animals need territories to increase their fitness during the breeding season. Dragonflies show elaborate territorial behavior (Corbet 1999). The mature male guards an area within a wetland and struggles with other males in order to maintain his territory and reproduce (Corbet 1999). By occupying it, the individual will reduce the limited resource, which is space, not only for conspecifics butalso for heterospecifics with similar morphological traits (color, size, or structural morphology) and similar habitat preferences. Such cases are common between congenerics (Warren and Lawton 1987; Juliano and Lawton 1990). In our study, it was supposed that the three species interacted regularly to own breeding territories. Since territories are valuable for territorial dragonflies during the breeding season, a close examination of one of the three main resources in species ecological niche proposed by Schoener (1974), namely the microhabitat.

One of the most common examples of habitat partitioning between sympatic congeneric species in ecological communities is that of Caribbean Lizards (*Anolis* spp), in which species use different perching heights and diameters within trees to gain spatial isolation (Schoener 1975; Losos 1994). During our study, the spatial distribution survey revealed that two species, *O. nitidinerve* and *O. chrysostigma,* mainly occupied open areas while the other one, *O. c. anceps,* occurred in highlyvegetated (covered) ones. Since niche overlap measure was based on microhabitat use, its value was highest between *O. nitidinerve* and *O. chrysostigma* (0.96), intermediate between *O. nitidinerve* and *O. c. anceps* (0.45), and lowest between *O. chrysostigma* and *O. c. anceps* (0.20). Thus, the two species with similar sizes and behaviors were spatially isolated.

Previous studies conducted on the subspecies *O. c. coerulescens* in Europe reported similar habitat preferences to that of *O. c. anceps,* with vegetated water as the typical suitable habitat (Heymer 1969), but the former subspecies is known to frequent other intermediate habitats (Buchwald 1989; Buchwald and Schmidt 1990; White 2008; Wildermuth 2008;). *O. nitidinerve* and *O. chrysostigma* had approximately the same habitat characteristics as those presented by Heymer (1969) for *O. brunneum.*

It is reasonable to think that sympatric congeneric species diverge at the microhabitat scale because they usually exploit local food in the same way (Benke and Benke 1975; Joahannsson 1978), which results in the exclusion of the less competitive. For example, in the eastern United States, two congeneric Zygopterans, *Enallagma aspersum* and *E. traviatum,* had different habitat preferences, with the former restricted to small fishless ponds and the latter to ponds with insectivorious fish (Pierce et al. 1985). Although spatial isolation of closely related species is the general pattern, other studies have shown that congeners and morphologically similar species converge in both time and space (Wissinger 1992; Crowley and Johnson 1982). In the current study, the two species with the same ecological preferences in terms of breeding territories interacted regularly, and the exclusion of *O. chrysostigma* by *O. nitidinerve* was thought to occur. Sokolovska et al. (2002) and Thompson and Fincke (2002) reviewed the effect of body size on territorial odonates' fitness, and they concluded that larger insects mate with more females (higher male mating rate) by winning more contests with other males. Since both species were phenotypically alike, the larger species (*O. nitidinerve*) might potentially win territorial contests more frequently. This could explain the low numbers of the latter species during the entire study period. In addition, three marked *O. chrysostigma* mature males were recovered in a channel at 300 m from the canal, but no individual male of the larger species was found to have displaced so far.

We inferred that both spatial heterogeneity and differential microhabitat preferences could maintain a mixed population of conge-neric species in the same system. We assume that similar patterns of microhabitat preferences might occur between the two congeneric species, *Orthetrum trinacria* and *O. sabina,* that coexist in the northern limits of the Algerian Sahara (Dijkstra et al. 2007). Our results suggest that the three studied species, especially *O. c. anceps,* were genetically predisposed to choose their respective microhabitats, as is usual in arthropods (Jaenike and Holt 1991). Further studies should focus on the larval stage to investigate if: (1) similar spatial patterns also occur during their aquatic life, (2) strong interspecific competition (exploitative or interference) or intraguild cannibalism occurs and to what extent, and 3) niche axes other than microhabitat act to maintain their coexistence.
